# Methicillin and Vancomycin Resistant *S. aureus* in Hospitalized Patients

**DOI:** 10.4103/0974-777X.68535

**Published:** 2010

**Authors:** Poonam Sood Loomba, Juhi Taneja, Bibhabati Mishra

**Affiliations:** *Department of Microbiology, G. B. Pant Hospital, New Delhi, India*

**Keywords:** Methicillin resistanc *Staphylococcus aureus*, Vancomycin resistance

## Abstract

*S. aureus* is the major bacterial cause of skin, soft tissue and bone infections, and one of the commonest causes of healthcare-associated bacteremia. Hospital-associated methicillin-resistant *S. aureus* (MRSA) carriage is associated with an increased risk of infection, morbidity and mortality. Screening of high-risk patients at the time of hospital admission and decolonization has proved to be an important factor in an effort to reduce nosocomial transmission. The electronic database Pub Med was searched for all the articles on “Establishment of MRSA and the emergence of vancomycin-resistant *S. aureus* (VRSA).” The search included case reports, case series and reviews. All the articles were cross-referenced to search for any more available articles. A total of 88 references were obtained. The studies showed a steady increase in the number of vancomycin-intermediate and vancomycin-resistant *S. aureus*. Extensive use of vancomycin creates a selective pressure that favors the outgrowth of rare, vancomycin-resistant clones leading to heterogenous vancomycin intermediate *S. aureus* hVISA clones, and eventually, with continued exposure, to a uniform population of vancomycin-intermediate *S. aureus* (VISA) clones. However, the criteria for identifying hVISA strains have not been standardized, complicating any determination of their clinical significance and role in treatment failures. The spread of MRSA from the hospital to the community, coupled with the emergence of VISA and VRSA, has become major concern among healthcare providers. Infection-control measures, reliable laboratory screening for resistance, appropriate antibiotic prescribing practices and avoidance of blanket treatment can prevent long-term emergence of resistance.

## INTRODUCTION

*S. aureus* is a leading cause of nosocomial infections, including bacteremia, surgical wound infections, as well as pneumonia.[1–3] About one quarter of healthy people carry one or more strains asymptomatically at any given time, and infections are commonly endogenous being caused by the patient’s colonizing strain.[4] Methicillin resistance was first detected in *S. aureus* in 1961,[5] shortly after the agent was introduced clinically; and over the last four decades, there has been a global epidemic of methicillin-resistant *S. aureus* (MRSA).[6,7] MRSA is usually acquired during exposure to hospitals and other healthcare facilities and causes a variety of serious healthcare-associated infections. The problem is exacerbated by the propensity of the organism to cause cross-infection and its ability to colonize individuals for months or years. Considerable selection pressure for this organism is applied in the hospital setting due to the now intensive use of the many antibiotics, particularly cephalosporins, to which the organism is resistant.

Vancomycin has been regarded as the first-line drug for treatment of MRSA. Unfortunately there has been an increase in the use of this antibiotic for other infections, such as pseudomembranous colitis due to *Clostridium difficile* and coagulase-negative staphylococcal infections in hospitalized patients.[8,9] When this drug was introduced in 1858, it was perceived that there would be no resistance to this antibiotic as resistance was very difficult to induce.[10] However, in 1997 the first stain of *S. aureus* with reduced susceptibility to vancomycin was reported from Japan.[11] Since then, there has been an increase in the number of cases with both VISA and VRSA (vancomycin-intermediate and vancomycin-resistant *S. aureus*). This has triggered off alarms in the medical community as *S. aureus* causes life-threatening infections in hospitalized and nonhospitalized patients.[12] The electronic database Pub Med was searched for all the articles “Establishment of MRSA and the emergence of VRSA.” The search included case reports, case series and reviews. All the articles were cross-referenced to search for any more available reports- yielding articles. Key words ‘methicillin resistant’ and ‘vancomycin-resistant *Staphylococcus aureus*’ yielded 16,888 articles. They were narrowed down when ‘epidemiology’ was added to the search term. The literature was also searched using ‘hospital-acquired MRSA and risk factors,’ ‘colonization and infection control,’ etc. This review will focus on the clinical, epidemiological and laboratory aspects of these infections.

## MECHANISM OF METHICILLIN RESISTANCE

Methicillin resistance is defined as the strains of *S. aureus* that are resistant to the isoxazoyl penicillins such as methicillin, oxacillin and flucloxacillin. MRSA are cross-resistant to all currently licensed β-lactam antibiotics.

The expression of methicillin resistance by *S. aureus* strains is by virtue of acquired penicillin binding proteinPBP2a, encoded by *mec* A gene.[13] Structurally, PBP2a possesses both transglycosylase and transpeptidase. PBP2a confer resistance to all β-lactam antibiotics. The origin of *mec* A gene is unknown. Expression of methicillin resistance in *S. aureus* is commonly under regulatory control by *mec* I or by *Bla* I gene. The *mec* I and *bla* I repressors are controlled by the *mec* RI and *bla* RI transducers. Expression of methicillin resistance in *S. aureus* is also influenced by the expression of other genetic loci called *fem* (“factors essential for methicillin resistance”) or *aux* (“auxiliary”) genes.[13] Many *fem* and *aux* factors have now been identified, which are involved in formation of the staphylococcal cell wall. The *mec* A gene is located within a larger region of chromosome known as the staphylococcal cassette chromosome *mec* (SCC*mec*) region (21-67 kb).[14] SCC*mec* is a mobile element, with mobility conferred by the presence of the *ccr*A and *ccr*B genes. The basic elements of SCC*mec* are the *mec*RI-*mec*I-*pbp*2a region and *ccr*A. Nosocomial isolates have larger SCC*mec*, owing to the accumulation over time of integrated plasmids or transposons that contribute to the multi-drug resistance.[14] There are five currently described SCCmec types (types I, II, III, IVa, IVb, V). Types I, II and III are found predominantly in healthcare-associated MRSA, whereas type IV is commonly found in the more susceptible community-associated MRSA.[15] Type IV SCC*mec* element is small and transferable by transduction. Types I to III SCC*mec* elements are large and hence do not transfer by bacteriophage. They are predominantly transferred by person-to-person spread of MRSA in the hospital rather than the spread of the resistant determinant from strain to strain. The spread of MRSA within institutions is therefore largely due to the transmission of resistant organisms from patient to patient, probably on the hands of transiently colonized healthcare workers.[16]

## DETECTION METHODS

Clinical and Laboratory Standards Institute (CLSI) recommends the cefoxitin-disk (30 µg) screen test, the latex agglutination test for PBP2a, or a plate containing 6 µg/mL of oxacillin in Mueller-Hinton agar supplemented with NaCl (4% w/v; 0.68 mol/L) as alternative methods of testing for MRSA and *mec* A detection based on PCR or hybridization. For *S. aureus*, the cefoxitin-disk (30 µg) test is comparable to the oxacillin-disk (1 µg) test for prediction of *mec* A–mediated resistance to oxacillin. However, cefoxitin is a better inducer of the mec A gene, and disk-diffusion test using cefoxitin gives clearer endpoints and is easier to read and thus is the preferred method than oxacillin.[17] The disk-diffusion method is reliable if incubation temperature is maintained at 35°C for 24 hours. Accurate detection of oxacillin/ methicillin resistance can be difficult due to the presence of two subpopulations (one susceptible and the other resistant) that may coexist within a culture of staphylococci, i.e., they are heteroresistant.[17] Each cell in the population may carry the genetic information for resistance, but only a fraction (10^-8^ to 10^-4^) can actually express the resistant phenotype under *in vitro* testing conditions. Cells expressing heteroresistance grow more slowly than the oxacillin-susceptible population and may be missed at temperatures above 35°C. The following tables show the breakpoints for defining methicillin resistance.[17]

## CLINICAL IMPORTANCE IN THE HOSPITAL

When patients with MRSA have been compared to patients with methicillin-susceptible *S. aureus* (MSSA), MRSA-colonized patients more frequently develop symptomatic infections.[18,19] Furthermore, higher case fatality rates have been observed for certain MRSA infections, including bacteremia, post-sternotomy mediastinitis and surgical-site infections.[20–27] Also some studies have reported an association between MRSA infections and increased length of stay, as well as healthcare costs, while others have not.[24,25,27]

Once MRSA is introduced into a healthcare setting, transmission and persistence of the resistant strain is determined by the availability of vulnerable patients, selective pressure exerted by antimicrobial use, increased potential for transmission from larger numbers of colonized or infected patients (“colonization pressure”), and the impact of implementation and adherence to prevention efforts.[28] Patients vulnerable to colonization and infection include those with severe disease, especially those with compromised host defenses from underlying medical conditions; recent surgery; or indwelling medical devices (e.g., urinary catheters or endotracheal tubes).[29] Hospitalized patients, especially ICU patients, tend to have more risk factors than nonhospitalized patients and have the highest infection rates. Studies have shown high prevalence (7%) of MRSA colonization at the time of patient admission.[30] MRSA acquisition has been shown to occur both in the healthcare setting and in the community.[30] A significant number of patients (2.2% of all adults admitted to the hospital) are colonized with community acquired CA-MRSA USA300 clone at the time of admission, which represents an emerging and increasingly problematic reservoir of MRSA in US hospitals.[31]

The emergence of new epidemic strains of MRSA in the community, among patients without established MRSA risk factors, may present new challenges to MRSA control in healthcare settings. Community stains of MRSA (e.g., USA300 and USA400) are being reported with increasing frequency within hospitals.[32,33] Changing resistance patterns of MRSA in ICUs in the National Nosocomial Infection Surveillance (NNIS) system from 1992 to 2003 provide additional evidence that the new epidemic MRSA strains are becoming established as healthcare-associated or community pathogens.[33] Infections with these strains have most commonly presented as skin disease in community settings. However, intrinsic virulence characteristics of the organisms can result in clinical manifestations similar to or potentially more severe than traditional healthcare-associated MRSA infections among hospitalized patients. The prevalence of MRSA colonization and infection in the surrounding community may therefore affect the selection of strategies for MRSA control in healthcare settings.

## GLOBAL EPIDEMIOLOGY OF MRSA

Bacterial strain typing distinguishes epidemiologically related or clonal isolates from unrelated isolates. Epidemiologically related isolates are viewed as descendants from a common precursor cell; thus, their genomic “fingerprints” will be indistinguishable but recognizably different from unrelated or random isolates from the same species.[34] In addition to tracking outbreaks, genotyping is used to distinguish between contaminating and infecting isolates and between separate episodes of infection and relapse of disease.[35]

Numerous techniques are available to differentiate *S. aureus*, and specifically MRSA, isolates. Historically, isolates were distinguished by phenotypic methods, including antibiotic susceptibility testing and bacteriophage typing. Both methods have limitations, as genetically unrelated isolates commonly have the same antibiogram, and many *S. aureus* isolates are nontypable by phage typing.[34]

With the advent of molecular biology, strain typing is focused on DNA-based methods. Initial techniques compared restriction endonuclease patterns of chromosomal or plasmidDNA. The second-generation of genotyping methods included s southern blot hybridization using gene-specific probes, ribotyping, polymerase chain reaction (PCR)–based approaches, and pulsed-field gel electrophoresis (PFGE).[36] These methods require subjective interpretation and comparison of patterns and fingerprint images. However, they still remain difficult to standardize between laboratories, and the image-based information is difficult to organize for rapid search and retrieval by computer. In addition, image-based methods do not provide biological criteria to evaluate the relatedness between different strains.[37] DNA sequence analysis is an objective genotyping method; the genetic code (A-T-C-G) is highly portable and easily stored and analyzed in a relational database. Recent advances in DNA-sequencing technology, including rapid, affordable, high-throughput systems, have made it possible for sequencing to be considered as a viable typing method. Two different strategies have been used to provide genotyping data: multilocus sequence typing (MLST), which compares sequence variation in numerous housekeeping gene targets; and single-locus sequence typing, which compares sequence variation of a single target among strains to be typed.

Two *S. aureus* genes conserved within the species, protein A (*spa*) and coagulase (*coa*), have variable short-sequence repeat (SSR) regions constructed from closely related 24- and 81-bp tandem repeat units, respectively. In both genes, the in-frame SSR units are degenerative, variable in number and variable in the order in which repeat units are organized. The genetic alterations in SSR regions include both point mutations and intragenic recombination that arise by slipped-strand mispairing during chromosomal replication and that result in a high degree of polymorphism.[38,39] DNA sequence analysis of the protein A repeat region provides an unambiguous, portable dataset that simplifies information-sharing between laboratories and facilitates creating a large-scale database for studying global and local epidemiology.[40]

Molecular epidemiology studies using different techniques indicate that the massive geographic spread of MRSA results from the dissemination of relatively few epidemic clones.[37,41,42] However, the “epidemic potential” depends on a multifactorial spectrum of bacterial genetic determinants, and the role the environment (selective usage of antibiotics, hygiene measures in the hospital) plays in their expression is unclear.

In 1999, MRSA accounted for >50% of *S. aureus* isolates from patients in ICUs in the NNIS system; in 2003, 59.5% of *S. aureus* isolates in NNIS ICUs were MRSA.[43] Prevalence of MRSA in hospitalized patients in south India has been shown to be 31.1%.[44] In Europe, the highest prevalence of MRSA in the hospitals was seen in Portugal (54%), Italy (43%-58%) and Netherlands (2%).[45]

## INFECTION-CONTROL ISSUES

The anterior nares are considered to be the primary colonization site, and approximately 30% of healthy people carry the bacteria in their anterior nares. Carrier rates close to 60% have been described previously for certain populations.[46] The throat has been considered as an important carriage site for *S. aureus*, although in lower numbers, and should be included when screening for *S. aureus*, including MRSA.[47] Multidrug-resistant organisms, such as MRSA or VRE, have been isolated from the hands, gloves, or both of HCWs involved in the care of infected or colonized patients.[48] Patient-to-patient transmission in healthcare settings, usually via hands of healthcare workers (HCWs), has been a major factor accounting for the increase in MRSA incidence and prevalence in acute-care facilities.[49] Preventing the emergence and transmission of these pathogens requires a comprehensive approach that includes administrative involvement and measures (e.g., nurse staffing, communication systems, performance-improvement processes to ensure adherence to recommended infection-control measures), education and training of medical and other healthcare personnel, judicious antibiotic use, comprehensive surveillance for MRSA, application of infection-control precautions during patient care, environmental measures (e.g., cleaning and disinfection of the patient-care environment and equipment, dedicated single-patient use of noncritical equipment) and decolonization therapy when appropriate. Screening for carriage of MRSA is fundamental for nosocomial infection control, both for epidemiological purposes and decisions on barrier isolation.

## THERAPEUTIC MEASURES

These include improvements in hand hygiene, use of contact precautions until patients are culture-negative for MRSA, active surveillance cultures, education, enhanced environmental cleaning and improvements in communication about patients with MRSA within and between healthcare facilities. In an effort to reduce nosocomial transmission of MRSA, surveillance cultures have been recommended at the time of hospital admission for patients at high risk of MRSA carriage.[49] Screening all patients admitted to a large institution can be logistically and financially challenging. Hence screening of patients at high risk of MRSA carriage is more practical. Studies have identified several risk factors for MRSA carriage at hospital admission, including prior receipt of antibiotic therapy, especially therapy with fluoroquinolones.[50] Decolonization entails treatment of persons colonized with MRSA, to eradicate carriage of that organism. Decolonization of persons carrying MRSA in their nares has proved possible with several regimens, which include topical mupirocin alone or in combination with orally administered antibiotics (e.g., rifampicin in combination with trimethoprim-sulfamethoxazole or ciprofloxacin) plus the use of an antimicrobial soap for bathing.[51] However, candidates receiving decolonization treatment must receive follow-up cultures to ensure eradication. It should be noted that recolonization with the same strain, initial colonization with a mupirocin-resistant strain, and emergence of resistance to mupirocin during treatment can occur.[52,53]

Healthcare personnel (HCP) implicated in transmission of MRSA are candidates for decolonization and should be treated and assured culture negative before returning to direct patient care. In contrast, HCP who are colonized with MRSA but are asymptomatic and have not been linked epidemiologically to transmission, do not require decolonization.

Contact precautions are intended to prevent transmission of MRSA which is transmitted by direct or indirect contact with the patient or the patient’s environment. HCP caring for patients on contact precautions should wear a gown and gloves for all interactions that may involve contact with the patient or potentially contaminated areas in the patient’s environment. If active surveillance cultures are used to detect and isolate patients colonized with MRSA or vancomycin resistant Enterococci VRE, and there is no decolonization of these patients, it is logical to assume that contact precautions would be used for the duration of stay in the setting where they were first implemented. In general, it seems reasonable to discontinue contact precautions when three or more surveillance cultures for MRSA are repeatedly negative over the course of a week or two in a patient who has not received antimicrobial therapy for several weeks. In several reports, cohorting of patients, cohorting of staff, use of designated beds or units and even unit closure were necessary to control transmission.[54–56]

Drugs approved for the treatment of MRSA infections are vancomycin, linezolid, daptomycin, teicoplanin, quinupristine-dalfopristine and tigecycline. The glycopeptide vancomycin has been regarded as the drug of choice for the treatment of infections due to methicillin-resistant strains.

## DEFINITION OF VANCOMYCIN RESISTANCE

There are different breakpoints used in defining vancomycin susceptibilities in different countries. This has led to confusion in the definitions and clinical significance of vancomycin resistance. According to the National Committee for Clinical Laboratory Standards (NCCLS), staphylococci for which MIC of vancomycin is ≤ 4 µg/mL are sensitive, while isolates for which MIC of vancomycin is 8-16 µg/mL are defined as intermediate sensitive (vancomycin-intermediate *S. aureus*, VISA). Strains having MIC of vancomycin ≥ 32 µg/mL are designated resistant (vancomycin-resistant *S. aureus*, VRSA). These guidelines are followed in US and Canada. In Japan, however, isolates with MIC 8 µg/mL are considered VRSA.[12] Heteroresistance was initially defined as the presence of >10^-6^ stable cell subpopulations of a strain that is apparently susceptible to vancomycin on the basis of conventional criteria, but for which the vancomycin MIC for the subpopulation of cells is greater than or equal to 8 mg/L.[57] According to Clinical and Laboratory Standards Institute (CLSI) breakpoints, hVRSA are strains of *S. aureus* containing subpopulations of vancomycin-intermediate daughter cells where the MICs for the parent strains of these daughter cells fall within the susceptible range of 1 to 4 µg/ml. The term GISA (glycopeptide-intermediate resistant *S. aureus*) may be more specific for strains intermediate sensitive for both vancomycin and teicoplanin, but not all VISA strains are intermediate sensitive to teicoplanin; so VISA is a more accurate term.

## EMERGENCE OF VANCOMYCIN RESISTANCE

### Heterogeneous VISA

The first case of heterogeneous VISA was reported in Japan in 1997 from a 62-year-old man with MRSA pneumonia who remained unresponsive after 12 days of vancomycin treatment.[58] Since then, a number of workers have conducted epidemiologic studies to determine the prevalence of this organism in their region. In a study in France, the prevalence of heterogeneous VISA was found to be 11%.[59] It has been reported worldwide from various countries, including Japan, Tehran, India, Korea,[60] Hong Kong,[61] Thailand,[62] Spain,[63] Greece,[64] Germany,[65] Italy[66] and the United Kingdom.[67] These authors have reported that the frequency of heterogeneous VISA in their publications was in the range of 0% to 74%.[68–70] Lui *et al*.,^69^ analyzed data from several studies and reported a prevalence of 1.64%. In most of these publications, surveillance was not performed routinely, the sample size was small, the studies were retrospective, or the methods for screening and identifying heterogeneous VISA varied among studies. Thus the interpretation of these studies is difficult.

### True VISA

True VISA was also reported in Japan in a 4-month-old infant in 1997.[11] Since then, more than 21 VISA strains have been isolated in the United States.[71]

Four cases of VRSA have been isolated in the US.[72] Vancomycin-resistant enterococci were also isolated in three of the four patients of VRSA, raising the possibility of transfer of vanA gene in these patients. While in many countries there are no strains with complete resistance to vancomycin, the major concern is the horizontal transfer between patients and establishment of endemic focus.[73]

### Predisposing factors and clinical significance of VRSA

Most of the strains have been isolated in hospitalized patients. However, there are cases reported even from the community. Environmental factors contributing to vancomycin resistance include irrational use of antibiotics; over-the-counter availability without prescriptions; injudicious use in hospitals, agriculture, fisheries and animal husbandry, which could result in increased selective pressure of vancomycin.[74] Among the clinical factors, exposure to glycopeptides or vancomycin is the biggest risk factor for VRSA and vancomycin-resistant coagulase negative staphylococci. Peritoneal dialysis and renal failure may also be risk factors.[75] The heteroresistant phenotype may be associated with treatment failure and/ or may be a precursor of glycopeptide resistance and should be considered in both empirical and rational therapy decisions.

The clinical significance of heterogeneous VISA is not clear. It is unknown whether levels of resistance are responsible for treatment failures or if these strains are as virulent as vancomycin-susceptible strains of *S. aureus*. It has been suggested that heterogeneous VISA strains are responsible for clinical failures to vancomycin treatment of otherwise apparently susceptible *S. aureus* strains.[64] Further studies are needed to evaluate the relevance of heterogeneous VISA in patients with clinical failure to vancomycin.

### Mechanism of vancomycin resistance

#### VISA

Vancomycin binds with the D-alanyl-D-alamine C terminus of the bacterial cell precursors, thereby preventing cross-linking by transpeptidation resulting in inhibition of cell wall production by attacking sites responsible for cell wall production.[76] VISA and hetero-VISA strains have been found to have thickened cell wall with reduced glycoprotein. This could be due to changes in peptidoglycan synthesis resulting in increased residues of D alanyl-D-alanine, which bind vancomycin molecules and prevent them from reaching the target sites.[77,78]

#### VRSA

VRSA strains also have been found to have thicker cell walls than the sensitive strains.[79] As with VISA strains, there is also increased peptidoglycan synthesis. It has been shown that vancomycin is only trapped in the outer layers and sequestered by the bacteria and not deactivated.[80,81] Exchange of genetic material is yet another mechanism postulated for VRSA. It has been suggested that patients at risk for VRSA are co-infected or co-colonized with VRE and MRSA, which enables transfer of vanA gene from VRE to MRSA in a biofilm environment leading to a VRSA strain.[82] In a case, it was reported that the patient had resistant *E. fecalis* in the wound, which caused the conjugative transfer of vanA gene.[83]

#### Coagulase negative staphylococci CONS

In coagulase-negative staphylococci, the exact mechanism is not known, but it has been noted that small amounts of altered cell wall precursors are produced and there are altered cross links.[84]

### Detection methods

Vancomycin resistance testing can be done by both automated and non-automated methods. Not all sensitivity testing systems detect VRSA. VRSA isolates are detected by reference broth microdilution, agar dilution, E test^®^, MicroScan^®^ overnight, BD Phoenix^™^ system, VRSA screen test for VITEK^®^ 2, Synergies plus^™^, TREK Sensititre MIC plate, disk diffusion and vancomycin screen agar plates (brain-heart infusion agar containing 6 µg/L vancomycin) (www.cdc.gov/ncidod/dhq/pdf/ar/visa_vrsa_guide.pdf). Non-automated methods for VISA detection include microdilution, agar dilution and E test. Disk-diffusion test does not detect VISA strains. VISA strains with vancomycin MIC 8 µg/L are detected by automated methods. Centers for Disease Control (CDC) have developed an algorithm for testing *S. aureus* (www.cdc.gov/ncidod/hip/vanco/vanco.htm). They state that the two acceptable primary test methods are (a) MIC method plus vancomycin VA screen plate and (b) disk diffusion and VA screen plate. Based on this, possible VISA and VRSA strains are identified. These isolates are re-tested to first reconfirm the purity and the genus and species of the organism, and then the result is verified by an MIC method (broth microdilution reference MIC, agar dilution, reference MIC or E test). CDC should then be notified.

Disk-diffusion sensitivity systems and automated methods are not very reliable in detecting VRSA. Disk diffusion using 30 µm/L disk does not identify intermediate-sensitivity isolates.[85] Two thirds of VRSA isolates were not identified by the MicroScan^®^ and VITEK^®^ systems according to a study by CDC.[86] The laboratories using automated methods must use a vancomycin agar screen plate in addition for testing of all MRSA isolates.

Testing for heteroresistant strains is also necessary. Many heteroresistant strains are unrecognized because the recommended screening methods present problems for diagnostic laboratories. The E test is the recommended test, but it is expensive if it is to be performed on all *S. aureus* isolates, and confirmatory testing with population analysis is labor intensive, time consuming and unsuitable for routine use.[87]

## CONCLUSION

There are only limited drugs available for the treatment of VRSA. Quinupristin-dalfopristin and linezolid are two of the newer antimicrobial agents currently available with activity against drug-resistant staphylococci (including most VISA and VRSA strains *in vitro*). Though cross-resistance has not been noted for linezolid, isolates have known to developresistance during therapy. Daptomycin, a bactericidal agent that damages the cytoplasmic membrane, is undergoing clinical trials.[78] Other agents in the pipeline include modified glycopeptides, carbapenems, oxazolidinones, quinolones and tetracyclines. But as they are still in the developmental stages, it will take almost a decade for new drugs to be launched. Avoiding irrational use of antibiotics and having rational antibiotic policy is the only way forward till then.

**Table:** Interpretive criteria (in µg/mL) for oxacillin MIC tests

	Susceptible	Intermediate	Resistant
*S. aureus*	≤ 2 µg/mL	N/A	≥ 4 µg/mL

**Table:** Interpretive criteria (in mm) for oxacillin disk-diffusion tests

	Susceptible	Intermediate	Resistant
*S. aureus*	≥ 13 mm	11–12 mm	≤ 10 mm

N/A - not applicable

**Table:** Interpretive criteria (in mm) for cefoxitin disk-diffusion test

	Susceptible*	†	Resistant**
*S. aureus*	≥ 22 mm		≤ 21 mm

* Report as oxacillin susceptible;

** Report as oxacillin resistant;

† There is no intermediate category with the cefoxitin disk-diffusion test
